# Phase I trial of Bermekimab with nanoliposomal irinotecan and 5-fluorouracil/folinic acid in advanced pancreatic ductal adenocarcinoma

**DOI:** 10.1038/s41598-022-19401-3

**Published:** 2022-09-02

**Authors:** Jun Gong, Shant Thomassian, Sungjin Kim, Gillian Gresham, Natalie Moshayedi, Jason Y. Ye, Julianne C. Yang, Jonathan P. Jacobs, Simon Lo, Nick Nissen, Srinivas Gaddam, Mourad Tighiouart, Arsen Osipov, Andrew Hendifar

**Affiliations:** 1 Department of Medicine, Samuel Oschin Comprehensive Cancer Institute, Cedars-Sinai Medical Center, Los Angeles, CA 90048 USA; 2 The Vatche and Tamar Manoukian Division of Digestive Diseases, Department of Medicine, David Geffen School of Medicine, University of California, Los Angeles, CA 90095 USA

**Keywords:** Drug safety, Clinical trial design, Drug development, Cancer, Cancer therapy, Gastrointestinal cancer, Pancreas, Gastrointestinal system, Microbiota

## Abstract

In this phase I dose-escalation trial, we assess the maximum tolerated dose (MTD) of Bermekimab in combination with Nanoliposomal Irinotecan (Nal-Iri) and 5-Fluorouracil/Folinic Acid (5-FU/FA). Secondarily, we investigate effects on weight, lean body mass, quality-of-life, the gut microbiome composition, inflammatory biomarkers, progression-free survival, and overall survival. This was a single-arm, open-label adaptive Bayesian dose-escalation study of Bermekimab combined with Nal-Iri and 5FU/FA in patients with advanced or locally advanced PDAC who failed gemcitabine-based chemotherapy. 22 patients enrolled between 2017 and 2019. 3 of 21 patients experienced dose-limiting toxicities attributable to the chemotherapy backbone. 58% (10/17) of patients exhibited weight stability. Physical performance status was preserved among all subjects. Patients reported improvements in quality-of-life metrics via QLQ-PAN26 questioner (−3.6, p = 0.18) and functional well-being (1.78, p = 0.02). Subjects exhibited a decrease in inflammatory cytokines, notably, vascular endothelial growth factor (−0.86, p = 0.017) with Bermekimab. Bermekimab treatment was associated with an increased abundance of gut health-promoting bacterial genera *Akkermansia,* with 3.82 Log2-fold change from baseline. In sum, Bermekimab is safe to be used in conjunction with Nal-Iri and 5-FU/FA chemotherapy. This benign toxicological profile warrants further Phase I/II investigation of Bermekimab in combinatorial strategies, and the impact of anti-IL-1α antibodies on the gut microbiome.

Clinical trials registration: NCT03207724 05/07/2017.

## Introduction

Pancreatic ductal adenocarcinoma (PDAC) has a 5-year survival of 10% and is projected to be the second leading cause of cancer mortality in the United States by 2030^1,2^. Unfortunately, the recent advances in precision medicine and immunotherapeutics are not applicable to most patients with PDAC. The molecular landscape of pancreatic cancer (PC) is governed by a preponderance of KRAS alterations (92%-93%), limiting the scope of targeted strategies in PC^3–13^. An exception is in KRAS wild-type patients where RAF alterations, NRG fusions, NTRK fusions are potentially actionable^3,5,6,14–16^. Immunotherapeutics have been generally unsuccessful as PDAC is characterized by low tumor mutation burden and lack of tumor infiltrating lymphocytes.

PDAC is also characterized by weight loss (WL) attributable to anorexia, malabsorption, and cancer cachexia^17^. At presentation, 80% of PDAC patients will have clinically significant weight loss and meet the definition of cancer cachexia^18^. Cachexia is a multifactorial syndrome characterized by progressive involuntary WL, loss of skeletal muscle mass, and systemic inflammation^19,20^. Weight loss and decreased lean body mass are associated with a poor prognosis, increased toxicity, post-op infections, and decreased therapeutic intensity^21–26^.

Systemic inflammation in PDAC has been linked to tumor proliferation and its associated cachexia^27,28^. Oncogenic KRAS activates STAT3 and Nuclear Factor-κB (NF-κB) by inflammatory cytokines, including Interleukin-1α, in an autocrine and paracrine manner leading to cancer initiation, metastases, and an inflammatory tumor micro-environment^29–31^. Elevations in c-reactive protein, in the neutrophil-to lymphocyte ratio, and pro-inflammatory cytokines such as TNF-alpha, IL-1α, and Interleukin-6 have been associated with pancreatic cancer cachexia, poor response to treatment, and poor outcomes such as decreased overall survival^27,32–35^.

Bermekimab (formerly known as MABp1) is a true human monoclonal antibody that specifically targets IL-1α. Bermekimab was cloned directly from a human B cell (Epstein-Barr virus-immortalized) that was isolated from a person with endogenous anti-IL-1α antibodies. In a phase 1 trial of solid tumor patients, there were no dose limiting toxicities and improvements in lean body mass were identified^36^. In a randomized phase 3 trial in colorectal cancer, Bermekimab treated patients were more like to reach a composite endpoint of stable or increased lean body mass and stability or improvement in two of three symptoms (pain, fatigue, or anorexia) at week 8^37^. The significance or reaching this novel endpoint was unclear, however these results warranted further development of IL-1α antagonism for the palliation of end-stage cancer patients^38,39^.

The present study aims to first establish the safety and the recommended phase 2 dose of the IL-1α antagonist (Bermekimab) in combination with Nanoliposomal Irinotecan (Nal-Iri) and 5-Fluorouracil (5FU)/Folinic Acid (FA) in patients with advanced pancreatic adenocarcinoma and cachexia who have failed gemcitabine-based chemotherapy. Secondarily, we aimed to assess the effects on weight, body composition, physical performance, and patient reported outcomes.

## Methods

### Patients

This was a single arm, single center, open-label phase I study of Bermekimab combined with Nal-Iri and 5FU/FA in patients with advanced or locally advanced PDAC who failed gemcitabine-based chemotherapy (includes gemcitabine-based combinatorial therapies and single-agent gemcitabine). Patients with histologically confirmed PDAC who were referred to the Cedars-Sinai Medical Center outpatient gastrointestinal (GI) oncology clinic for systemic therapy were screened. Key inclusion criteria included: advanced or locally advanced PDAC that has progressed through or intolerant of gemcitabine-based chemotherapy, cachexia defined as > 5% unexplained weight loss 6 months prior to screening or as documented by physician, Eastern Cooperative Oncology Group (ECOG) performance status (PS) 0–2 or Karnofsky Performance Scale (KPS) ≥ 60%, and normal organ and marrow function. The study experimental protocol was approved by the Cedars-Sinai Medical Center Institutional Review Board, and written informed consent was obtained from each patient and/or their legal guardians before enrollment. All research was performed in accordance with relevant guidelines/regulations.

### Study design

**Figure 1:**
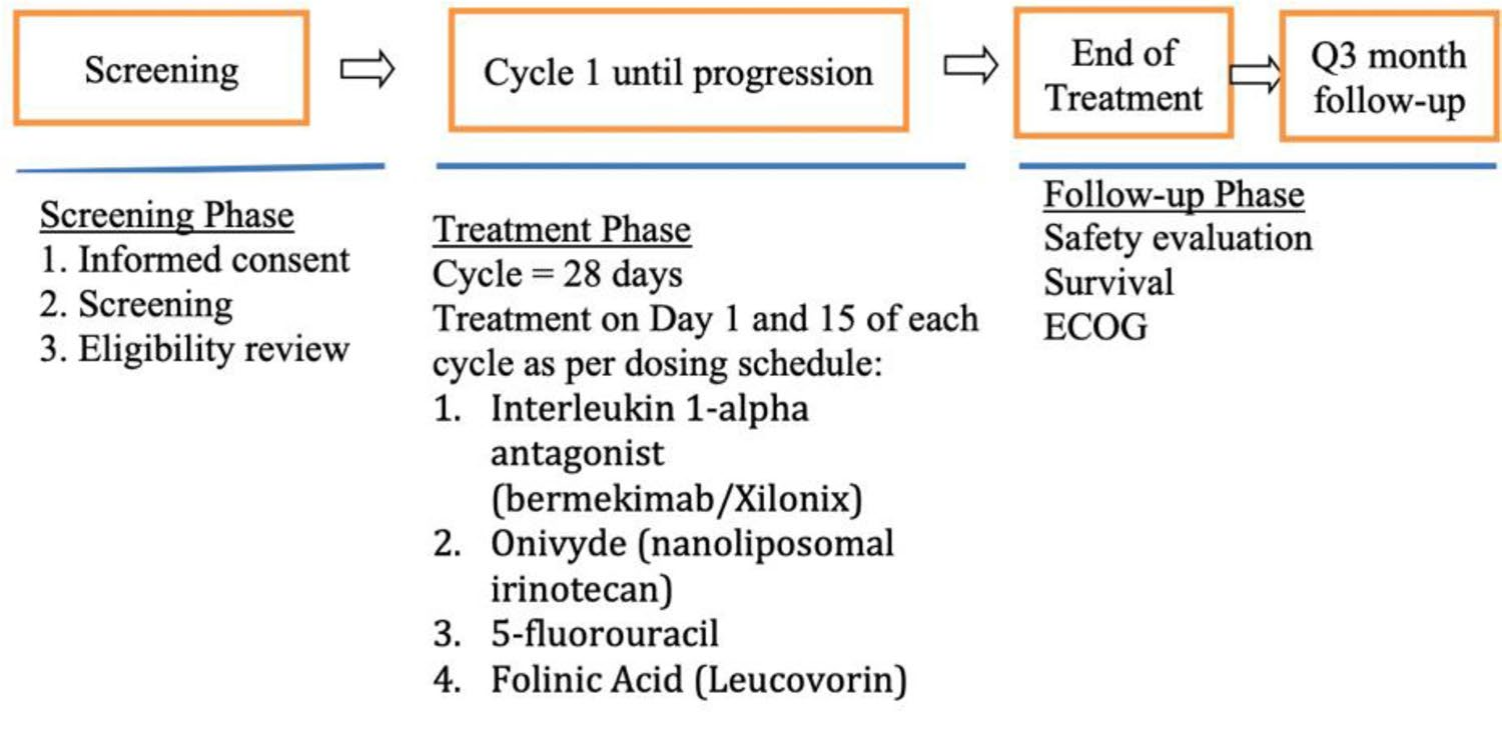
Study schema.

The primary objective was to determine the maximum tolerated dose (MTD) of Bermekimab in combination with Nal-Iri and 5FU/FA using Bayesian adaptive design based on dose-limiting toxicities (DLTs) in the first cycle. The MTD was defined as the dose such that the probability of DLTs at MTD is θ = 0.33. Dose levels of Bermekimab at 3.75 mg/kg, 7.5 mg/kg, or 12.0 mg/kg intravenous (IV) every other week, Nal-Iri at 50 mg/m^2^ or 70 mg/m^2^ IV day 1 and 15, and 5FU 2000 mg/m^2^ or 2400 mg/m^2^ day 1 and 15, every 4-week cycles, were employed for the dose finding schema. Initially, the trial was designed to explore four dose levels described in Table 1 and a new dose level 3 would be added by increasing the dose of Bermekimab to 12.0 mg/kg if there is statistical evidence that the probability of DLT at dose level 2 is below the target θ by 10%, using the extended escalation with overdose control (EWOC) algorithm with a flexible range of doses described in Tighiouart et al. 2018^40^. Specifically, if after treating *n* patients using EWOC algorithm, if *P*( P(DLT|dose = level 2) < *θ* – 0.1 | data) > 0.8, then dose level 3 would be introduced. Operating characteristics of the design can be found in the clinical protocol (Fig. 1).

**Table 1:** Dose escalation schedule.

Dose level	Dose			
	Xilo Xilonix IV (mg/kg)	5-Fluorouracil (5FU) 2400 mg/m^2^	Folinic acid (Leucovorin) 400 mg/m^2^	Onivyde IV 70 mg/m^2^
Level −2	3.75 mg/kg	2000 mg/m^2^	400 mg/m^2^	50 mg/m^2^
Level −1	7.5 mg/kg	2000 mg/m^2^	400 mg/m^2^	50 mg/m^2^
Level 1	7.5 mg/kg	2400 mg/m^2^	400 mg/m^2^	50 mg/m^2^
Level 2	7.5 mg/kg	2400 mg/m^2^	400 mg/m^2^	70 mg/m^2^
Level 3	12.0 mg/kg	2400 mg/m^2^	400 mg/m^2^	70 mg/m^2^

FA doses were fixed at 400 mg/m2 IV. The first cohort of up to three patients received dose level -1 and the subsequent doses were determined by the escalation with overdose control (EWOC) algorithm^40^. As part of this study, participants did not receive any additional nutrition support or concomitant therapies.

Weight stability, defined as weight change < 0.1 kg per baseline BMI-unit was evaluated at 2 months from baseline. DEXA scans—a bone density assessment—were utilized to assess changes in lean body mass at 2 months from baseline. Grip strength (measured via dynamometer), gait speed, daily activity and sleep (measured continuously via wrist-worn biometric sensor, Fitbit Charge 3) were used as markers to track patient performance. Quality of life measures were self-reported by patients via EORTC QLQ-PAN26 and FAACT questionnaires. Levels of inflammatory cytokines were evaluated for up to a 12-month period from baseline. Survival data including overall survival from date of diagnosis and progression free survival were collected for all patients.

Additionally, gut microbiome was analyzed by using stool samples prospectively collected at predefined time points “pre-intervention” (C1D1) and “post intervention” (C3D1). DNA extraction and sequencing of the 16S ribosomal RNA gene was performed for fecal samples as previously described^41^. Bacterial DNA was extracted using the ZymoBIOMICS DNA kit (cat#D4300) with bead beating. The V4 region of the 16S gene was amplified and barcoded using 515f/806r primers then 250 × 2 bp sequencing was performed on an Illumina MiSeq system. Raw data were processed using DADA2 scripts in R platform and quality-filtered reads (mean 58,814 reads per sample) were used to identify amplicon sequence variants (ASV) with taxonomy assigned using the Silva database^42^.

### Statistical analysis

All patients who received any amount of the study drug were evaluable for safety (per CTCAE version 4.03). After 13 patients were evaluable for DLT, the risk that the probability of DLT at dose level 2 being less than 23% was equal to 0.84, i.e., *P*(*P*(DLT|dose = level 2) < *θ* – 0.1 | data) = 0.84, therefore dose level 3 was introduced. At the end of the trial, the MTD was estimated as the median of the posterior distribution of the MTD given data on all evaluable patients, and dose level 3 was recommended as the MTD. Efficacy including overall response rates were assessed as per RECIST 1.1 criteria. Demographics, baseline characteristics, and safety variables were summarized by descriptive statistics; categorical variables were reported as frequency (percentage, %) and continuous variables were reported as mean (standard deviation, SD) or median (interquartile range, IQR). Paired sample t-test or Wilcoxon signed-rank test was used, as appropriate, to examine differences in lean body mass, grip strength, gait speed, and daily activity and sleep from trial initiation to 2 months (cycle 3). Changes in inflammatory cytokines markers over 12 months from trial initiation were examined using a generalized additive model for location, scale and shape (GAMLSS) with patient as a random effect and using a log normal distribution with an identity link function^43^. The goodness of fit of a model was examined using residuals such that the most adequate response distribution is chosen^44^. P-values were adjusted for multiple tests using the Holm procedure. Progression-free survival (PFS) was defined as time in months from the date of consent to progressive disease or death. Overall survival (OS) was defined as time in months from the date of consent to death. Survival functions were estimated by the Kaplan–Meier method^45^. Analyses were performed using R Statistical Software (v4.0.5; R Core Team 2020) with two-sided tests and controlling for the family-wise error rate of 0.05.

Microbiome alpha diversity was assessed using the Shannon and Chao1 indices on data rarefied to 33,151 sequences/sample. Significance of differences in alpha diversity pre- vs. post- intervention were determined by paired t-test. Differentially abundant taxa (filtered to remove those with less than 25% prevalence) were identified using DESeq2, which implements a Bayesian approach towards shrinking dispersion and fitting non-rarified abundance counts to a negative binomial model. Measurements of differential relative abundance in terms of log2-fold change were generated while retaining the measure of normalized mean relative abundance as a proportion of total estimated counts of taxa. P-values for differential abundance were adjusted by the Benjamini–Hochberg method to control false discovery rate (Padj < 0.05 was considered significant).

## Results

Twenty-two eligible patients were enrolled on study with informed consent between October 17th, 2017, and October 24th, 2019. Gender was evenly represented in the subject population with 11 males and 11 females, with a median age 67 (range 61–75) and median BMI of 21.17 kg/m^2^ (range 19.32–23.42 kg/m^2^). Of the 22 patients, 12 identified as Non-Hispanic Caucasian (54.55%), 5 as Non-Hispanic Asian/Other (22.73%), 3 as Hispanic (13.64%), and 2 African American (9.09%). Among the patients 13 reported never smoking, 7 were former smokers, and 2 were continued active smokers. All patients had ECOG performance status of ≤ 1; 20 patients with ECOG of 1.

Toxicity data is available for 21 of the 22 patents enrolled as 1 patient withdrew consent prior to receiving study treatment dose. Based on prespecified criteria in which patients had completed more than two cycles or 4 weeks of this novel treatment, 16 patients were evaluable for DLTs. Among all patients enrolled, only three experienced DLTs, which were deemed unrelated to Bermekimab. DLTs included: grade 3 fatigue, vomiting, diarrhea, and anorexia. All-grade toxicities associated with the chemotherapy backbone of Nal-Iri and 5-fluorouracil included diarrhea (71%), fatigue (71%), nausea (57%), and vomiting (52%). Of these, grade 3/4 toxicities are as follows: 1 subject with diarrhea (5%), 5 subjects with fatigue (24%), 2 nausea (10%), and 3 with vomiting (14%) (Table 2). Due to the statistical analysis of the DLT, a dose level 3 was recommended as the MTD in this treatment combination.

**Table 2:** Dose limiting toxicities (DLT) in cohort.

DLT (grade 3 or 4)	Number of subjects (%)
Diarrhea	1 (5%)
Fatigue	5 (24%)
Nausea	2 (10%)
Vomiting	3 (14%)

**Figure 2:**
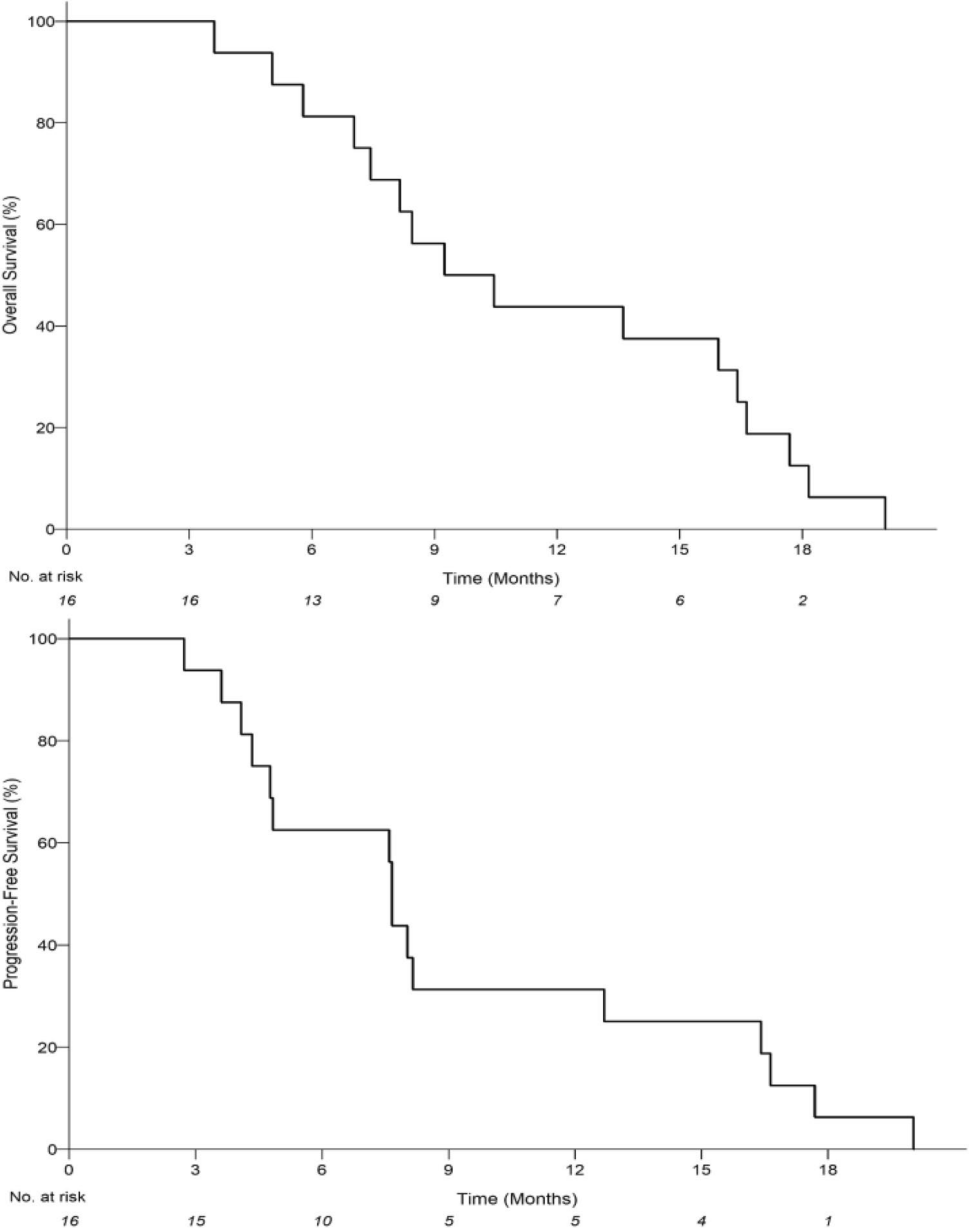
Kaplan–Meier estimates of the probability of progression-free survival (PFS) for evaluable patients receiving intervention. Median PFS is 7.66 months (95% CI 4.34–12.70) with an estimated 12-month PFS rate of 31.25% (95% CI: 11.39–53.65) (top). Kaplan–Meier estimates of the probability of overall survival (OS) for evaluable patients receiving intervention. Median OS is 9.85 months (95% CI 7.04–16.41) with an estimated 12-month OS rate of 43.75% (95% CI 19.81–65.56) (bottom).

Among evaluable subjects, 5/16 patients had a partial response to treatment. The median progression-free survival (PFS) was 7.66 months (95% CI 4.34–12.70) and the median overall survival (OS) was 9.85 months (95% CI 7.04–16.41) (Fig. 2). In the per protocol analysis, the response rate was 5/22 with median PFS 7.66 months (95% CI 4.08- 8.45), and OS 8.45 months (95% CI 5.79–13.62).

### Weight stability and body composition

DEXA measurements of body composition and BMI for cycles 1 and 3 were available in 17 of the 22 subjects (Table 3) and used to evaluate weight stability. Weight stability was achieved in 10/17 (58.8%) subjects at 2 months from baseline. Despite many patients demonstrating stable weight, throughout the study, they exhibited significant decrease in lean body mass and fat mass between cycles 1–3 (8 weeks). Mean change in lean body mass was −1.64 kg (± SD, 2.01; p = 0.003). Mean change in fat body mass was −1.37 kg (± 1.75; p = 0.004). Changes in bone mineral density were not significant but trend in a similar direction (median: −0.01 g/cm^2^; IQR: −0.03 to 0; p = 0.072).

**Table 3:** Body composition from DEXA scan.

Variable	Cycle 1	Cycle 3	Change	P-value
Total body bone mineral density (g/cm^2^)	1.11 (0.98–1.18)	1.05 (0.95–1.19)	−0.01 (−0.03 to 0)	0.072
Total body fat mass (kg)	16.65 (± 8.10)	15.49 (± 7.84)	−1.37 (± 1.75)	0.004
Total body fat (%)	28.34 (± 9.19)	27.16 (± 9.99)	−0.89 (± 2.02)	0.077
Lean body mass (kg)	41.07 (± 9.03)	40.32 (± 8.52)	−1.64 (± 2.01)	0.003

Data are presented as mean (± standard deviation) or median (interquartile range) as appropriate.

P-value is calculated by paired sample t-test or Wilcoxon signed-rank test as appropriate.

17 patients are included in analysis due to missing data at cycle 1 and/or cycle 3.

### Patient performance

Patients did not experience any significant changes in performance status between cycles 1–3 (Table 4). At trial initiation (cycle 1), mean handgrip strength in dominant hand measured 25.1 kg (± 9.2), 10-m walk was 5.2 s (± 1.4), Fitbit step count was 2703.3 (± 1912.9) per day, and resting heart rate was 68.8 bpm (± 9.3). Respectively, by the end of cycle 3 hand grip strength measured 24.9 (± 6.6), 10-m walk was 5.5 (± 1.8), Fitbit step count was 3200 (± 2304.8), and resting heart rate was 62.6 bmp (± 5.9).

**Table 4:** Patient performance.

Variable	Cycle 1	Cycle 3	Change	N	P-value
Hand grip strength (dominant) (kg)	25.12 (± 9.15)	24.86 (± 6.59)	0.27 (± 2.72)	11	0.747
Hand grip strength (other hand) (kg)	23.65 (± 8.11)	23.08 (± 5.82)	– 0.2 (± 3.63)	10	0.866
Walking speed (seconds)	5.2 (± 1.44)	5.45 (± 1.8)	0.01 (± 1.32)	8	0.992
Fitbit step count	2703.32 (± 1912.88)	3199.83 (± 2304.77)	107.18 (± 2143.36)	8	0.892
Fitbit heart rate	68.83 (± 9.28)	62.63 (± 5.88)	– 3 (± 3.9)	6	0.118

*N* sample size. 6–11 patients are included in analysis due to missing data at Cycle 1 or 3.

Data are presented as mean (± standard deviation).

P-value is calculated by paired sample t-test.

### Quality of life and patient-reported fatigue and physical function

QLQ-PAN26 scores were received from 15 patients. In paired sample t-test comparing measures at baseline and cycle 3 patients show improvements in total score with mean total scores of 56.5 and 52.9 (−3.6, p = 0.18). In assessing PAN26 scores aggregated into subscales, patients showed significant improvements in the hepatic subscale (−0.27, p = 0.04), with a trend toward improvements in pancreatic pain (−3.6, p = 0.18), body image (−0.2, p = 0.64), healthcare satisfaction (−0.87, p = 0.14), and sexuality (−0.61, p = 0.31). While there were no changes in the digestive subscale (0, p = 1.0), we found worsening in altered bowel habit, which was not statistically significant (0.73, p = 0.07).

FAACT questionnaire scores are available for 19 patients. There was no significant change in total scores from baseline through cycle 3, with mean total scores of 77.8 and 78.6 respectively. Paired T-tests for FAACT subscales showed a significant improvement in functional well-being (1.78, p = 0.02) with no significant changes in physical well-being (−1.2, p = 0.78), emotional well-being (−1.7, p = 0.14), social well-being (−0.57, p = 0.8), or additional concerns (−0.12, p = 0.93).

### Cytokines and biomarkers of interest

Estimated effects of intervention with Bermekimab in addition to chemotherapy over the first 6 months was associated with a significant decrease in various assessed cytokines. Levels of VEGF are found to be significantly decreased in cycle 3 (p-value = 0.009) and cycle 7 (p-value = 0.005) when compared to cycle 1 (reference cycle). Intervention also showed significant effects in IL-1RA in cycles 3, 5, and 7. Additionally, IL-6 and IL-4 where found do be reduced in cycle 7 vs. cycle 1 (p-value = 0.019) (supplemental Fig. 1). IL-1α and GM-CSF show a significant decrease in biomarker populations at cycle 7 when compared to cycle 1. Levels of leptin were reduced significantly at cycle 3 when compared to cycle 1 (supplemental Fig. 2). We did not find significant changes to soluble CD40 ligand (CD40L) (p-value = 0.110), epidermal growth factor (EGF) (p-value = 0.078), fibroblast growth factor -2 (FGF-2) (p-value = 0.096).

### Effects on microbiome and taxonomy

Stool was collected from 14 patients at baseline (C1D1) and post intervention (C3D1) totaling 28 samples. There was no significant difference in microbial alpha diversity after intervention by the Chao1 index of richness (138.6 pre- vs. 122.1 post-intervention, p = 0.51) and the Shannon index of richness and evenness (3.98 pre- vs. 3.85 post-intervention, p = 0.82). Differentially abundant taxa at the genus level were identified by DESeq2 (Table 5). Notably, *Akkermansia* was one of two differentially abundant genera and had a significant Log2 fold change of 3.82 post-intervention (Padj = 0.033).

**Table 5:** DESeqQ2 genus level data comparing post-intervention to pre-intervention.

Genus	Relative abundance	Log2 fold change	Standard error	Padj
*Erysipelatoclostridium*	0.0046	−8.75	1.79	6.6E−05
*Akkermansia*	0.017	3.82	1.16	0.033

**Figure 3:**
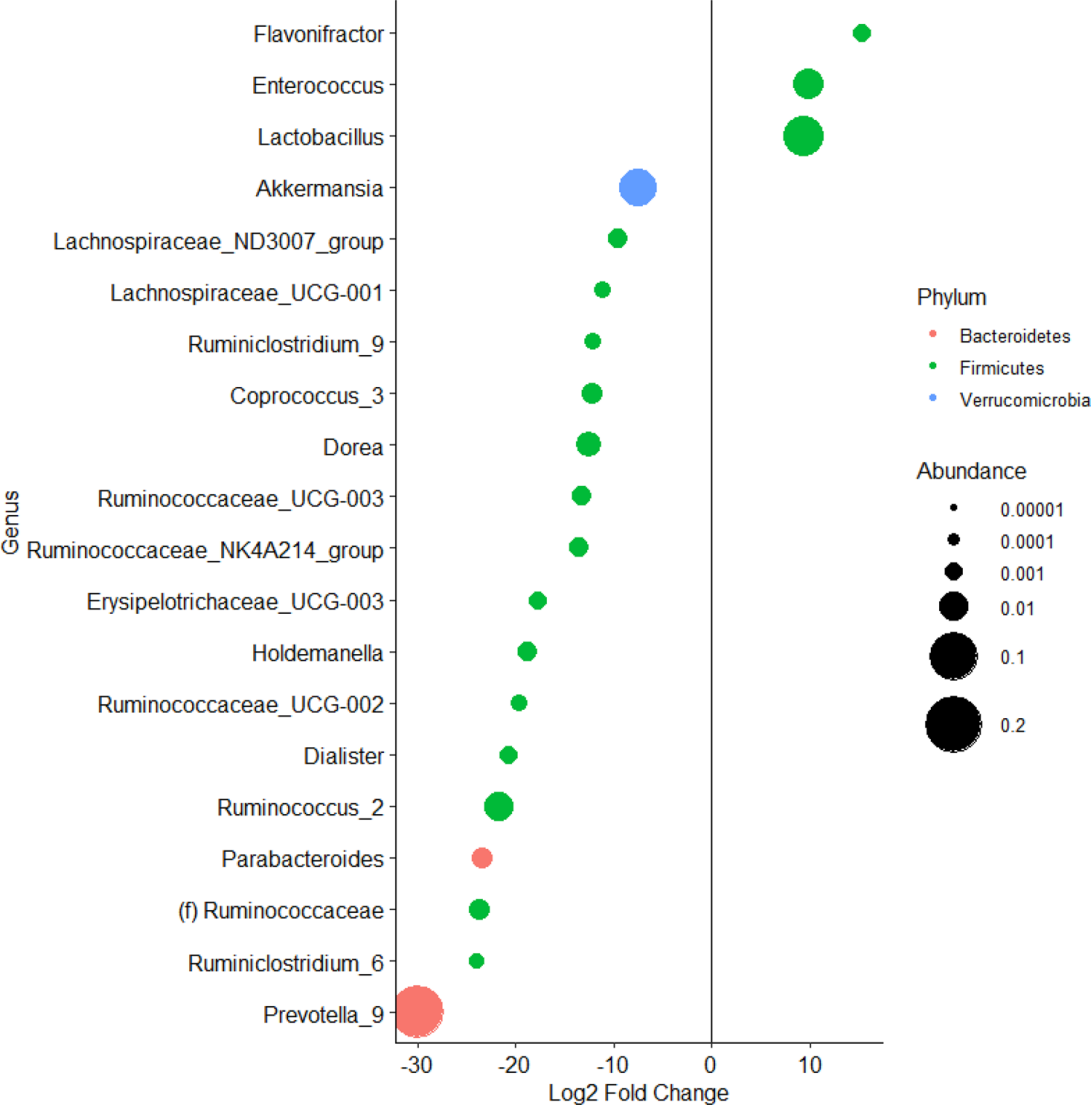
Plot showing Log2(fold-change) of bacterial genera abundance in samples from patients with increased IL-1α levels post-intervention compared to samples from patients with reduced IL-1α levels post-intervention. Each dot represents one bacterial genus and is colored by phylum and sized according to average relative abundance.

DESeq2 analysis was then performed at the genus level to compare post-intervention samples from patients with increased IL-1α levels post-intervention to samples from patients with reduced IL-1α levels post-intervention. This revealed a greater relative abundance of *Akkermansia* in samples from patients with reduced IL-1α post-intervention and in addition to significant differences in 19 other bacterial genera (Fig. 3). Linear regression did not demonstrate a significant association between the numerical levels of IL-1α and log-transformed counts of *Akkermansia* (estimate = −0.0074, SE = 0.017, t = −0.44, Pr( >|t|) = 0.67).

## Discussion

This phase-1 trial assessed the safety of the addition of an antibody to IL-1α to the standard chemotherapy regimen of 5FU and Nal-Iri in patients with locally advanced or advanced PDAC who had failed or were intolerant to gemcitabine-based chemotherapy. The combination was found to be safe, and tolerability was excellent with only three DLTs in 22 patients enrolled. When addressing the primary objective of determining the MTD in this novel therapeutic combination, this study was successful and the MTD in this cohort was a dose level 3. The regimen also demonstrated promising tumor response activity and overall survival in a cohort of refractory pancreatic cancer patients with associated cachexia. The median OS in second line trials of pancreatic cancer is 6.1–6.3 months for 5FU based combination therapies^46^. In our cohort, evaluable patients had a median OS of 9.8 months and the per protocol analysis had a median OS of 8.5 months. Compared to the NAPOLI-1 trial of Nal-Iri compared to 5FU/FA, Nal-Iri demonstrated an objective response rate of 16%^47^. Out of 16 evaluable subjects in this study, we observed a promising five partial responses (31%) with the combination of Nal-Iri and Bermekimab.

In this dose escalation study, we did not encounter DLTs attributable to the addition of Bermekimab. Of the 22 patients enrolled, three experienced DLTs which include grade 3 fatigue, vomiting, diarrhea, and anorexia. However, these DLTs are thought to be associated with the chemotherapeutic backbone. Overall, the regimen was well tolerated in a group of refractory pancreatic cancer patients. However, fatigue was found in 24% of participants, which compares unfavorably to the landmark NAPOLI-1 trial. This could be explained by the inclusion of only cachectic cancer patients but should be examined in more detail in future studies.

When compared with the NAPOLI-1 trial, where 5FU and Nal-Iri was compared to 5FU alone, the toxicity experienced in this phase 1 combination trial was generally favorable^47^. There were no appreciable differences in hematologic toxicity with all grade anemia and neutropenia being similar between 30 and 40% in both cohorts and thrombocytopenia did not occur in either study. All grade fatigue and anorexia were also common across both cohorts. Although all-grade diarrhea was common in our cohort 15/21 (71%) grade 3/4 diarrhea was uncommon 1/21 (5%) and not dose limiting. In the NAPOLI-1 trial, gastrointestinal toxicity resulted in dose reductions for 33% of patients receiving the combination of 5-fluorouracil and Nal-Iri.

Chemotherapy associated intestinal mucositis is currently understood to include the generation of reactive oxygen species, activation of nuclear factor-κB (NF-κB) and upregulation of proinflammatory cytokines including tumor necrosis factor (TNF), IL-6, and IL1b^48^. In mouse models of 5-fluorouracil induced intestinal mucositis, administration of IL-1Ra eliminated severe diarrhea and accelerated body weight recovery by blocking the destructive effect of IL-1β and reducing apoptosis in the small intestinal crypt after chemotherapy^49,50^.

Additionally, we identified a novel finding in that Bermekimab treatment was associated with greater abundance of *Akkermansia* in gut microbiome analyses. *Akkermansia*, particularly *Akkermansia* *muciniphila*, is a well-characterized microbe that plays a central role in promoting gut health and maintaining integrity of the gut barrier^51,52^. *A.* *muciniphila* regulates mucin production, a major component of the mucous layer that serves as the protective lining of the gastrointestinal mucosa. Disruption of the gut barrier results in “leaky gut” and diffusion of immune-activating substances that can result in systemic inflammation with implications across a host of metabolic diseases and cancer. Through regulation of the mucin layer and production of active metabolites such as short-chain fatty acids (SCFAs), *A.* *muciniphila* has been shown to reduce the risk of obesity, improve glucose tolerance, modulate immune responses, reduce systemic inflammation, and improve lipid metabolism^51,52^. Notably, there are growing efforts to increase abundance of *A.* *muciniphila* through diet and probiotics, fecal microbiata transplantation, and antimetabolic drugs to enhance the efficacy of metabolic and cancer therapies^51,52^. We are among the first to show that treatment over time with Bermekimab, which targets IL-1α, was associated with higher abundance of *Akkermansia*. We also demonstrated that patients with a post-treatment reduction in circulating IL-1α levels exhibited higher *Akkermansia* abundance than those without IL-1α reduction, in addition to higher abundance of 14 other genera belonging to phylum Firmicutes, among which *Ruminococcus* and *Dialister* are prominent SCFA producers^52^. This data supports a microbiome shift corresponding to the reduction of IL-1α following treatment with Bermekimab. These findings, in addition to our remarkably lower levels of G3/4 gastrointestinal toxicity in this cohort compared to NAPOLI-1, are hypothesis-generating and warrant further investigation into the Bermekimab and *Akkermansia* relationship, particularly as a means to ameliorate chemotherapy associated-diarrhea and promote gut health to mitigate systemic inflammation in randomized trials.

Notably, we observed significant reductions in vascular endothelial growth factor (VEGF) levels over time with combination Nal-Iri, 5FU/FA, and Bermekimab. This is a novel finding and uncovers an additional mechanism of action to potentially exploit with Bermekimab. VEGF signaling is considered a canonical angiogenesis promoter for multiple tumors whereby targeting VEGF has been established broadly in the treatment landscape of oncology from other gastrointestinal cancers such as colorectal and hepatocellular carcinoma to genitourinary cancers such as renal cell carcinoma where combination of anti-VEGF therapies with immunotherapy are foundational treatments. As such, it would be prudent to further examine the anticancer mechanisms of Bermekimab and anti-IL-1α therapy through its potential as an anti-angiogenic. Given the exquisite safety and tolerability of Bermekimab with no real overlapping toxicities with many systemic anticancer agents commonly used, Bermekimab remains an attractive agent for combinatorial strategies across multiple tumor types in this regard.

Weight stability in this cohort of refractory patients was common and encouraging. Despite the majority (10/17) experiencing weight stability, there were significant decrements in both lean body mass and fat body mass based on body composition analysis. This is in contrast to another phase 1 trial of anti-IL-1α monotherapy in treatment-refractory solid tumor patients, where lean body mass increased in 30 patients; the majority of whom were losing weight in the 6 months before enrollment^36^. Fat mass in this study did decrease as well by a median of −4.4% but of note, only 2 (4%) patients in this trial had PDAC. Presence of IL-1α monocytes in peripheral blood decreased over time with anti-IL-1α monotherapy, which was used as a pharmacodynamic measure in this study. We similarly observed a decrease in IL-1α levels over time, but notably, IL-1RA levels and IL-4 levels decreased as well. IL-1 receptor signaling has been shown to mediate cachexia, but IL-1RA is a natural inhibitor of the pro-inflammatory effect and has been used to treat certain autoimmune conditions such as rheumatoid arthritis^36,53^. IL-4 is generally thought to be an anti-inflammatory cytokine as well^54^. We observed a decrease in IL-6 levels over time with combination treatment in our cohort, which historically has been elevated in cancers that induce cachexia^55^. Therefore, although we observed decreases in IL-1α, IL-4, and IL-6 that would putatively promote an anti-inflammatory phenotype and mitigate cachexia, it is unclear whether the counter-effect of decreased IL-1RA plays a role in such discrepancies with lean body mass across studies.

Body composition analysis is not commonly described in prospective studies of refractory pancreatic cancer patients receiving an intervention. We recently conducted a prospective observational study evaluating the impact of enteral tube feeding on weight stability and body composition in a similar cohort of pancreatic cancer patients with cachexia. Weight stability was achieved and increases in lean body mass and appendicular lean mass were observed as well as decreases in fat mass in this small sample of patients who completed three cycles of tube feeding^56^. However, this study was limited by small sample size and heterogeneity in the chemotherapy regimen used. Still, findings from this study, taken together with findings from our Bermekimab trial, suggest alterations in gut microbiome secondary to chemotherapy and nutritional intervention may benefit weight preservation in advanced cancer. This warrants further investigation into each of these mechanisms to characterize patients suffering from cachexia who would stand to benefit most from one approach over another.

Additionally, previously reported retrospective studies have demonstrated that loss of lean body mass and fat mass are common among PDAC patients, independent of body mass index (BMI), associated with poor outcomes^57,58^. Notably loss of lean body mass has also been correlated with increased dose limiting toxicities and neuropathy for PDAC patients receiving gemcitabine and nab-paclitaxel^59^.

In our cohort, our measures for patient physical performance were stable over a 12-week period (C1–3). Hand-grip strength, step count, and walking speed were notably unchanged despite general loss of lean body mass in this cohort. These measures have not been similarly assessed in a refractory pancreatic cancer protocol and therefore it is difficult to benchmark our findings. However, these measures were easy to implement in our outpatient clinic and the information gathered could help evaluate the effects of our interventions—intended or otherwise. We have previously noted that physical function and the autonomy to perform daily and social activities is highly prioritized in pancreatic cancer patients^60^. Further development of our measures for patient performance are therefore needed.

## Conclusion

In this phase I trial, the addition of Bermekimab (formerly known as MABp1), a monoclonal antibody that specifically targets IL-1α, to Nal-Iri and 5FU/FA in advanced PDAC patients who have previously been treated with gemcitabine-based therapies demonstrated promising efficacy and was well tolerated. We observed weight stability in over half of the patients and changes in select cytokines involved with inflammatory cascades and the cachexia process that are hypothesis generating and warrant further understanding. Notably, study treatment significantly reduced VEGF levels uncovering a mechanism to exploit as a potentially novel anti-angiogenic with Bermekimab. This is provocative given the safety and tolerability of this drug and the fact that multiple tumor types have been targetable through VEGF blockade as monotherapy or in combination systemic therapies. The relationship between Bermekimab, targeting IL-1α, and promotion of gut health through impact on the gut microbiome also warrant further study.

## Supplementary Information

Supplementary Figure 1.

Supplementary Figure 2.

## Data Availability

All individual participant data collected during the trial, after deidentification will be shared with investigators who’s proposed use of the data has been approved by an independent review committee identified for this purpose. Data sets along with Study protocol will be available beginning 3 months and ending 5 years following article publication. responding author on reasonable request. Proposals should be directed to Andrew.Hendifar@cshs.org. To gain access, data requestors will need to sign a data access agreement.
