# Author Correction: Phase I trial of Bermekimab with nanoliposomal irinotecan and 5‑fuorouracil/folinic acid in advanced pancreatic ductal adenocarcinoma

**DOI:** 10.1038/s41598-022-23619-6

**Published:** 2022-11-04

**Authors:** Jun Gong, Shant Thomassian, Sungjin Kim, Gillian Gresham, Natalie Moshayedi, Jason Y. Ye, Julianne C. Yang, Jonathan P. Jacobs, Simon Lo, Nick Nissen, Srinivas Gaddam, Mourad Tighiouart, Arsen Osipov, Andrew Hendifar

**Affiliations:** 1 Department of Medicine, Samuel Oschin Comprehensive Cancer Institute, Cedars-Sinai Medical Center, Los Angeles, CA 90048 USA; 2 The Vatche and Tamar Manoukian Division of Digestive Diseases, Department of Medicine, David Geffen School of Medicine, University of California, Los Angeles, CA 90095 USA

Correction to: *Scientific Reports* 10.1038/s41598-022-19401-3, published online 02 September 2022

The Acknowledgments section in the original version of this Article was omitted. The Acknowledgments section now reads:

“This work is partially funded by NIH the National Center for Advancing Translational Sciences (NCATS) UCLACTSI (UL1 TR001881-01), NCI grant P01CA233452-02, and U01 grantCA232859-01.”

The original Article has been corrected.

