# Supplementary material for: Phase I trial of Bermekimab with nanoliposomal irinotecan and 5-fluorouracil/folinic acid in advanced pancreatic ductal adenocarcinoma
Source: Sci Rep. 2022 Sep 2;12:15013. doi: 10.1038/s41598-022-19401-3 (PMC9440135; doi:10.1038/s41598-022-19401-3)

*Supplemental Figure 1: Estimated effects of intervention on VEGF (top left), IL-1RA (top right), IL-6 (bottom left), and IL-4 (bottom right) with 95% Confidence Interval. Levels of VEGF are found to be significantly decreased in cycle 3 (p-value=0.009) and cycle 7 (p-value=0.005) when compared to cycle 1 (reference cycle). Intervention also shows significant effects in IL-1RA in cycles 3, 5, and 7. Additionally, IL-6 and IL-4 where found do be reduced in cycle 7 vs. cycle 1 (p-value=0.019).*


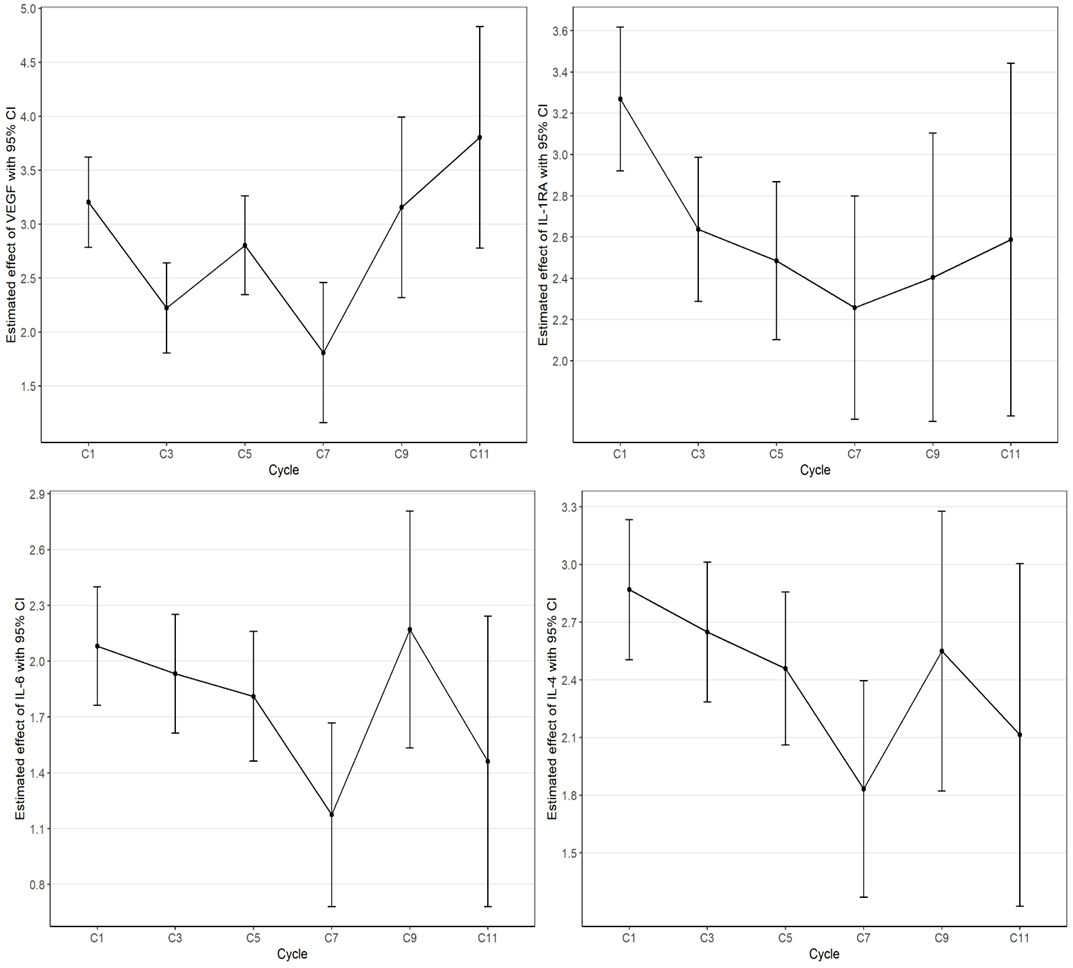

Supplement: Supplementary file 1 — Supplementary Figure 1. [file 41598_2022_19401_MOESM1_ESM.docx]
