# Supplementary material for: Phase I trial of Bermekimab with nanoliposomal irinotecan and 5-fluorouracil/folinic acid in advanced pancreatic ductal adenocarcinoma
Source: Sci Rep. 2022 Sep 2;12:15013. doi: 10.1038/s41598-022-19401-3 (PMC9440135; doi:10.1038/s41598-022-19401-3)

*Supplemental Figure 2: Estimated effects of intervention on IL-1α (top left), GM-CSF (top right), and Leptin (bottom) with 95% confidence interval. IL-1α and GM-CSF (top left and top right) show a significant decrease in biomarker populations at cycle 7 when compared to cycle 1. Levels of leptin were reduced significantly at cycle 3 when compared to cycle 1.*


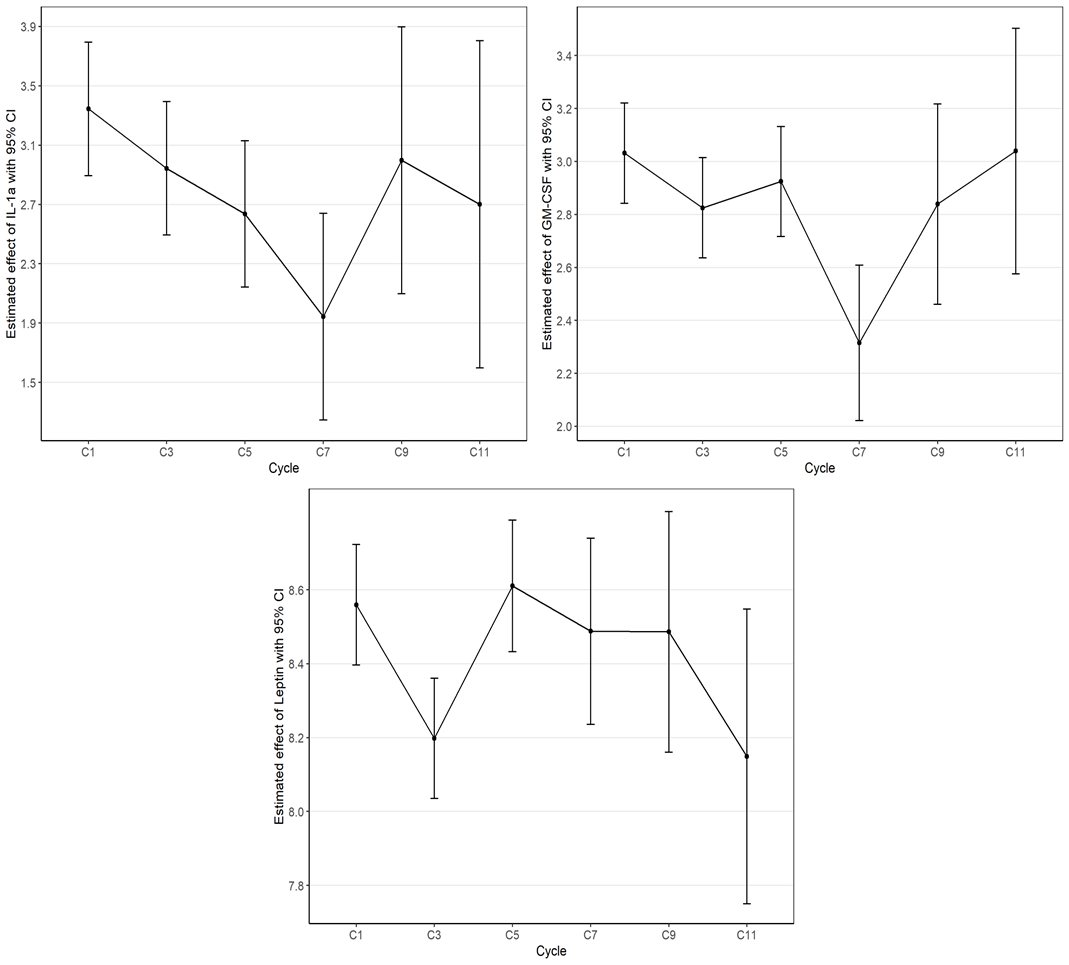

Supplement: Supplementary file 2 — Supplementary Figure 2. [file 41598_2022_19401_MOESM2_ESM.docx]
